# Intermittent Fasting Modulation of the Diabetic Syndrome in Streptozotocin-Injected Rats

**DOI:** 10.1155/2012/962012

**Published:** 2012-01-12

**Authors:** Louiza Belkacemi, Ghalem Selselet-Attou, Emeline Hupkens, Evrard Nguidjoe, Karim Louchami, Abdullah Sener, Willy J. Malaisse

**Affiliations:** ^1^Laboratoire de Technologie Alimentaire et Nutrition, Université de Mostaganem, 1070 Mostaganem, Algeria; ^2^Laboratory of Experimental Hormonology, Université Libre de Bruxelles, 808 Route de Lennik, 1070 Brussels, Belgium; ^3^Laboratory of Pharmacology, Université Libre de Bruxelles, 808 Route de Lennik, 1070 Brussels, Belgium

## Abstract

This study investigates the effects of intermittent overnight fasting in streptozotocin-induced diabetic rats (STZ rats). Over 30 days, groups of 5-6 control or STZ rats were allowed free food access, starved overnight, or exposed to a restricted food supply comparable to that ingested by the intermittently fasting animals. Intermittent fasting improved glucose tolerance, increased plasma insulin, and lowered Homeostatis Model Assessment index. Caloric restriction failed to cause such beneficial effects. The *β*-cell mass, as well as individual *β*-cell and islet area, was higher in intermittently fasting than in nonfasting STZ rats, whilst the percentage of apoptotic *β*-cells appeared lower in the former than latter STZ rats. In the calorie-restricted STZ rats, comparable findings were restricted to individual islet area and percentage of apoptotic cells. Hence, it is proposed that intermittent fasting could represent a possible approach to prevent or minimize disturbances of glucose homeostasis in human subjects.

## 1. Introduction

Overabundant food intake with chronic positive energy balance leads to obesity and type 2 diabetes, whilst reduction in food intake, by increasing insulin sensitivity and improving glucose homeostasis, is currently recommended in the treatment of these metabolic disorders [1–4]. Such a caloric restriction may include a relative decrease of food intake [5–7] or otherwise either a total short [8, 9] or prolonged [10] fasting.

Intermittent overnight fasting, inspired by the daily fasting period during the Ramadan, was recently reported to prevent the progressive deterioration of glucose tolerance otherwise taking place in sand rats exposed to a hypercaloric diet [11–13]. The major aim of the present study was to investigate whether a comparable benefit of intermittent fasting may prevail in streptozotocin-induced diabetic rats.

## 2. Materials and Methods

### 2.1. Streptozotocin-Induced Diabetes

Eight to 10 weeks after birth, female Wistar rats (Charles River, Wilmington, MA, USA) were injected intraperitoneally, after overnight starvation, with streptozotocin (STZ, 65 mg/kg body wt.) freshly dissolved in a citrate buffer (50 mM, pH 4.5). These rats were given access during the night after the injection of streptozotocin to a solution of saccharose (10 g/100 mL) to prevent possible hypoglycemia. Control rats were injected with the citrate buffer. Five days after the injection of streptozotocin, the glycemia was measured with the help of glucometer (Lifescan Benelux, Beerse, Belgium) in blood obtained from caudal vein. Only those rats displaying a glycemia in excess of 16.7 mM were kept for further investigations.

### 2.2. Starvation and Restricted Food Supply

In order to compare the effects of an intermittent fasting, mimicking the Ramadan fasting, to that of a caloric restriction, the experimentation in both control and STZ rats was conducted over two successive periods. Twenty days after the injection of either streptozotocin (STZ rats) or the citrate buffer vehicle (control rats), the rats were either given free access to food throughout the experimental period (NF: nonfasting rats), deprived of food and water from 5 p.m. to 8 a.m. (IF: intermittently fasting rats) or given access from 5 p.m. onwards to an amount of food comparable to that ingested by the IF rats (CR: calorie-restricted rats). Relative to the food intake in NF rats, such a caloric restriction represented a 20% decrease in food intake in the control animals and a 40% decrease of food intake in the STZ rats.

### 2.3. Body Weight and Food Intake

The initial body weight was measured before the injection of streptozotocin or its citrate buffer vehicle, 7 days thereafter, 20 days thereafter, on day 4, 7, 11, 14, 18, 21, and 27 of the final 30 days experimental period and at sacrifice, after overnight starvation. Likewise, food intake was measured 15 to 20 days (6 measurements) after injection of STZ or its vehicle in 6 control rats and 3 groups of 5-6 STZ rats, and daily (26 measurements) during the last 30 days experimental period.

### 2.4. Intraperitoneal Glucose Tolerance Test (IPGTT)

An IPGTT [14, 15] was conducted in all rats on day 10, 20, and 29 of the final 30 days experimental period, after overnight starvation. A solution of D-glucose (20%, w/v) in distilled H_2_O was intraperitoneally injected in conscious rats in order to deliver 2 g D-glucose per kg body weight. The glycemia was measured by a glucometer before and 30, 60, and 120 min after the administration of D-glucose in blood samples obtained from a caudal vein. The total and incremental areas under the glycemic curve (AUC) were computed in each individual experiment.

### 2.5. Sacrifice

At the end of the experimental period, the rats were sacrificed after overnight starvation and under anesthesia provoked by the intraperitoneal injection of a solution containing ketamine and xylocaine. Blood samples were obtained from the heart and placed in heparinized tubes, the plasma being then separated by centrifugation and stored at −80°C. The plasma D-glucose [16] and insulin [17] concentrations were measured by methods described in the cited references. These measurements were used to calculate the insulinogenic index (i.e., the ratio between the plasma insulin concentration, expressed as mU/L, and the difference between the plasma D-glucose concentration, expressed as mM, and 4.0 mM, considered as the threshold value for stimulation of insulin secretion by the hexose) and the HOMA index (i.e., the product of the plasma insulin concentration, expressed as *μ*U/mL, times the plasma D-glucose concentration, expressed as mM). The pancreas were either used for the isolation of islets or fixed for immunohistochemical examination.

### 2.6. Insulin Secretion In Vitro

Groups of 4 islets each, obtained by the collagenase procedure [18], were incubated at 37°C for 90 min in 0.5 mL of a salt-balanced medium [19] containing bovine serum albumin (5 mg/mL) and equilibrated against a mixture of O_2_/CO_2_ (95/5, v/v). The insulin released by the islets during incubation and their final insulin content were measured by radioimmunoassay [17].

### 2.7. Immunohistochemical Study

For immunodetection of insulin, pancreatic rehydrated paraffin sections were blocked 1 h at room temperature with 1 : 20 normal goat serum (Vector Laboratories, Burlingame, CA, USA) in PBS for nonspecific reactions. The slides were incubated with primary anti-insulin (12018, Sigma-Aldrich, St Louis, MO, USA) mouse monoclonal antibody overnight at 4°C at a concentration of 1/3000 in normal goat serum (1/20 in PBS). The secondary antibody, Rhodamine Red X-conjugated goat anti-mouse IgG (H + L) (115-295-146, Jackson ImmunoResearch Laboratories, West Grove, PA, USA) was applied at a dilution of 1/200 in PBS/normal goat serum for 30 min at room temperature. The slides were mounted, and DNA was counterstained with DAPI (In Vitrogen, Merelbeke, Belgium). The staining patterns were observed with an Axioplan and recorded with an Axiocam (Carl Zeiss, Oberkochen, Germany).

### 2.8. *β*-Cell Mass Assessment

Pancreatic sections were stained for insulin using standard ABC-DAB technique [20]. The slides were incubated overnight at 4°C with the first antibody: anti-insulin (Santa Cruz Biotechnology, Inc., CA, USA) at dilution 1 : 500 in PBS with appropriate blocking serum at a dilution of 1/20. Purified immunoglobulins (IgG) (Sigma-Aldrich, St Louis, MO, USA) from nonimmunized rabbit were used as negative controls. The slides were further incubated with the secondary biotinylated antibody: goat anti-rabbit IgG (H+L) (BA-1000, Vector Laboratories) at a dilution of 1/300 in PBS for 30 min, at room temperature. *β*-cell mass was measured by point-counting morphometry on these immunoperoxidase-stained sections [21]. The measurement was performed on live using Leica Microsystems microscope (Heerbrugg, Switzerland). A grill of 110 points was used to assess insulin positive stained islet on each field.

### 2.9. Individual *β*-Cell Area

Individual *β*-cell area was determined by using image J logician on immunofluorescence stained sections of pancreas used for *β*-cell apoptosis assessment. The *β*-cell area was calculated from the ratio between individual area and *β*-cell nuclei number within the area taken in consideration.

### 2.10. Glucagon Immunodetection

For glucagon immunodetection, the same procedure as that described for insulin immunodetection by the ABC-DAB technique [20] was used. The sole difference consisted in the first antibody, that is, antiglucagon (A0565, Dako, Carpinteria, CA, USA) used at dilution 1 : 400.

### 2.11. Apoptosis Detection

The quantification of *β*-cell apoptosis by the TUNEL method was performed using the *in situ Cell Death Detection kit*, POD (Roche Diagnostics, Vilvorde, Belgium). At the end of this procedure, the pancreatic sections were rinsed with PBS and eventually exposed overnight at 4°C to primary anti-insulin antibody (see above) followed by exposition for 30 min at 20°C to the Rhodamine Red X-conjugated secondary antibody (1/200 dilution). The apoptotic index represents the ratio between positive and total nuclei of insulin-producing cells in each islet.

### 2.12. Presentation of Results

All results are presented as mean values (±SEM), together with the number of separate determinations *(n)*. The statistical significance of differences between mean values was assessed by use of Student's *t*-test.

## 3. Results

### 3.1. Body Weight

**Figure 1:**
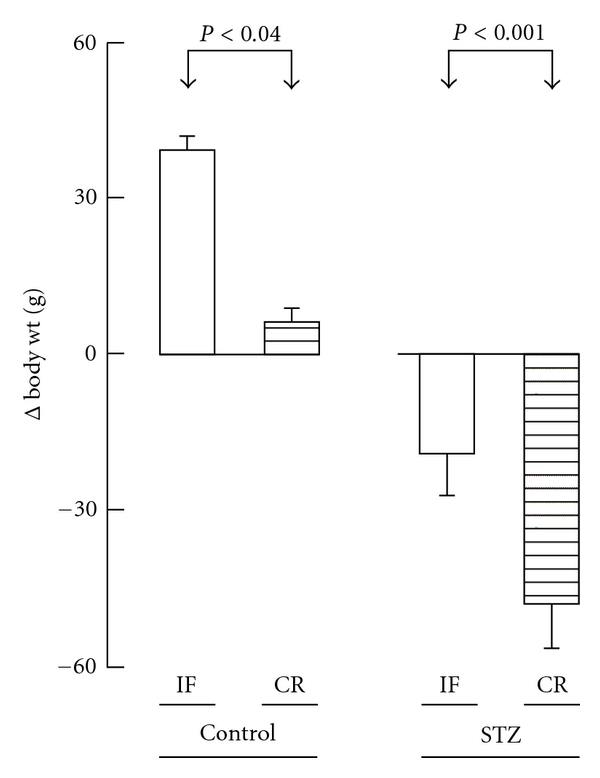
Comparison between the changes in body weight over the last 30 days experimental period in IF and CR control or streptozotocin rats. Mean values (±SEM) refer to 5-6 individual measurements.

At day zero of the last 30 days experimental period, the mean body weights of the control and STZ rats did not differ significantly (*P* > 0.52) from one another, with an overall mean value of 223 ± 4 g (*n* = 33). Over the 2 weeks following the injection of either streptozotocin or the citrate buffer vehicle, the changes in body weight averaged +17.6 ± 4.0 g (*n* = 16) in control rats, as distinct (*P* < 0.005) from −2.0 ± 5.0 g (*n* = 17) in STZ rats. Over the last 30 days of the experiments, the changes in body weight failed to differ significantly, whether in the control or STZ rats, when comparing IF animals to NF animals, with overall mean values of +33.8 ± 2.7 g (*n* = 10) in the control rats and −15.9 ± 4.4 g (*n* = 11) in the STZ rats. Expressed as a daily change in body weight, these two mean values were not significantly different (*P* > 0.4 or more) from those recorded in the same type of rats (control or STZ) over the 2-week period following the injection of streptozotocin or the citrate buffer vehicle. Over the last 30 days of the experiments, the gain in body weight was much lower (*P* < 0.04 or less) in the CR animals than in the IF animals, with mean values of +6.0 ± 3.1 g (*n* = 6) in control rats and −47.5 ± 8.7 g (*n* = 6) in STZ rats (Figure 1). A comparable situation (*P* < 0.07 or less) prevailed when considering the changes in body weight over the entire experimental period. 

### 3.2. Food Intake

As indicated in Table 1, the food intake over the last 6 days of the control period (day 15 to day 20 after the injection of STZ or its solvent), was more than twice higher (*P* < 0.001) in the STZ rats than in the control animals. Such a difference persisted when comparing fed control and STZ rats over the 30 days experimental period. In the IF and CR rats examined during the last 30 days experimental period, the food intake was again almost twice higher (*P* < 0.001) in STZ rats than in control animals.

The individual values considered in Table 1 represented the mean of 6–26 measurements in each rat. During the last 6 days of the control period, the variation coefficient (SD/mean) for the 6 successive measurements made in each rat amounted to 7.3 ± 0.6% (*n* = 23) in control and STZ rats. Likewise, over the last 30 days of the present experiments, the variation coefficient for the 26 measurements made during this period averaged 9.9 ± 0.8% (*n* = 5) and 9.1 ± 1.1% (*n* = 5) in NF control and STZ rats, respectively, as compared (*P* < 0.001) to 15.5 ± 0.3% (*n* = 5) and 16.5 ± 0.3% (*n* = 6) in IF control and STZ rats.

### 3.3. IPGTT

The paired difference between the glycemia at min 30 and min zero of the IPGTT was comparable (*P* > 0.77) in control rats (6.31 ± 0.62 mM; *n* = 48) and STZ rats (6.68 ± 1.15 mM; *n* = 52).

**Figure 2:**
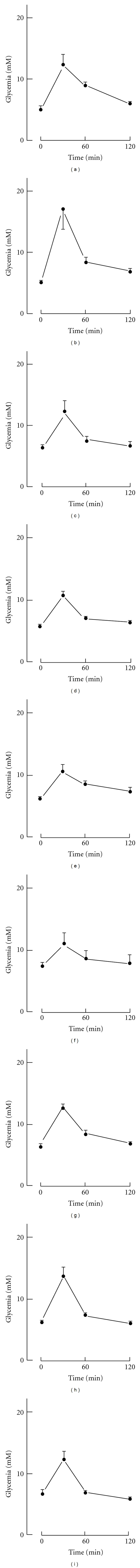
Glycemic profile during IPGTT conducted on day 10 (left), 20 (middle), and 29 (right) of the final experimental period in NF (upper panels), IF (middle panels), and CR (lower panels) control rats. Mean values (±SEM) refer to 5-6 individual experiments.

 The profile of glycemia during the IPGTT conducted in control rats is illustrated in Figure 2. The total AUC averaged in the IF and CR control rats, respectively, 94.0 ± 3.9% (*n* = 16) and 96.1 ± 2.8% (*n* = 17) of the mean corresponding values recorded on the same day in the fed control rats (100.0 ± 5.0%; *n* = 15). None of these mean values differed significantly from one another (*P* > 0.35 or more). However, as documented by the data listed in Table 2, the incremental AUC tended to be lower in IF and CR control rats than in fed control rats. Thus, the values recorded in IF and CR control rats, respectively, averaged 59.1 ± 15.3% (*n* = 16) and 65.3 ± 9.5% (*n* = 17) of the mean corresponding values recorded on the same day in the fed control rats (100.0 ± 15.1%; *n* = 15). Such a difference only achieved statistical significance (*P* < 0.04) when comparing the overall mean value recorded in both IF and CR rats (62.3 ± 9.5%; *n* = 33) to that found in the fed control rats.

**Figure 3:**
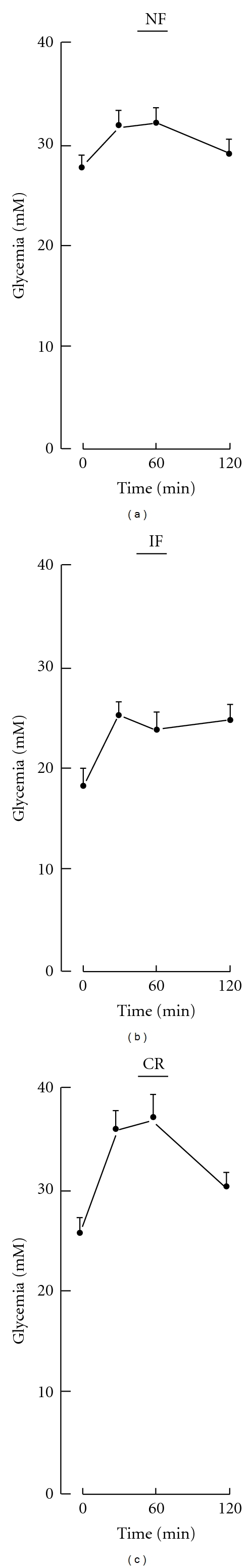
Glycemic profile during IPGTT conducted in NF (a), IF (b), and CR (c) STZ rats. Mean values (±SEM) refer to 15–20 individual experiments.

In the STZ rats, the results of the IPGTT were closely comparable in 4 groups of NF animals examined 20 days after the injection of streptozotocin or on day 10, 20, and 29 of the final experimental period. Hence, these results were pooled together. Likewise, the results of the IPGTT conducted on day 10, 20, and 29 of the final experimental period were pooled together in either the IF or CR STZ rats (Figure 3). The time zero glycemia was lower (*P* < 0.007 or less) in IF rats than in either NF or CR rats, no significant difference (*P* > 0.24) being observed between the latter two groups of STZ rats (Table 3). Likewise, the total AUC was lower (*P* < 0.001) in IF rats than in either NF or CR rats, which failed to differ significantly (*P* > 0.21) from one another. The incremental area, however, was not significantly different (*P* > 0.08 or more) in IF rats, as compared to either NF or CR rats, being only significantly higher (*P* < 0.02) in the CR rats than in the NF diabetic rats.

### 3.4. Plasma D-Glucose, Insulin Concentrations, and Insulinogenic and HOMA Indices at Sacrifice

The plasma D-glucose concentration was about 3-4 times higher in STZ rats than in control rats (Table 4). In the control rats, it was comparable (*P* > 0.49 or more) in NF, IF, and CR animals (Table 4). In the STZ rats, however, the overall mean value found in the IF and CR animals (24.63 ± 2.91; *n* = 12) was significantly lower (*P* < 0.04) than that recorded in the NF STZ rats.

 The plasma insulin concentration was about twice lower in STZ rats than in control animals. In the latter animals, the mean values recorded in either the IF or CR animals did not differ significantly (*P* > 0.26 or more) from that found in the NF animals. Such was also the case (*P* > 0.15 or more) in the STZ rats.

**Figure 4:**
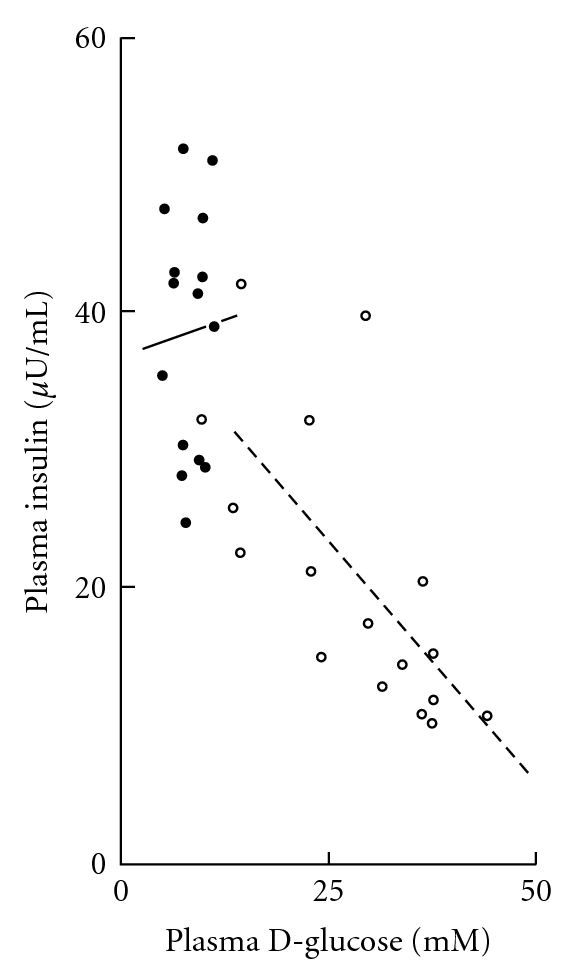
Comparison between plasma insulin and D-glucose concentrations found at sacrifice in control (closed circles and solid line) and STZ (open circles and dashed line) rats. The two oblique lines correspond to the regression lines.

As illustrated in Figure 4, no significant correlation was observed between plasma insulin and D-glucose concentration in the 15 control animals (*r* = +0.0416; *P* > 0.1), whilst a highly significant negative correlation between these two variables prevailed in the 17 STZ rats (*r* = −0.6892; *P* < 0.004). Covariance analysis, however, indicated that the two regression lines failed to differ significantly from one another in either their slope (*F* = 0.567; *f* = 1,28; *P* > 0.25) or elevation (*F* = 2.156; *f* = 1,28; *P* > 0.1).

The insulinogenic index was much higher (*P* < 0.001) in control rats (10.60 ± 1.84 mU/mmol; *n* = 15) than in STZ animals (0.90 ± 0.21 mU/mmol; *n* = 17). In each of these two sets of rats, no significant difference was found between NF, IF or CR animals. At the most, there was a trend (*P* < 0.09) towards a higher value for the insulinogenic index in IF and CR diabetic rats (1.17 ± 0.34 mU/mmol; *n* = 12) than in the NF STZ rats (0.48 ± 0.08 mU/mmol; *n* = 5).

 The HOMA index for insulin resistance did not differ significantly in the 3 groups of control rats, with an overall mean value of 312 ± 35 mM·*μ*u/mL (*n* = 15). Such was also the case in the STZ rats, with an overall mean value of 518 ± 51 mM·*μ*u/mL (*n* = 17) significantly higher (*P* < 0.004) than that recorded in the control animals.

### 3.5. Pancreatic Islet Data

**Figure 5:**
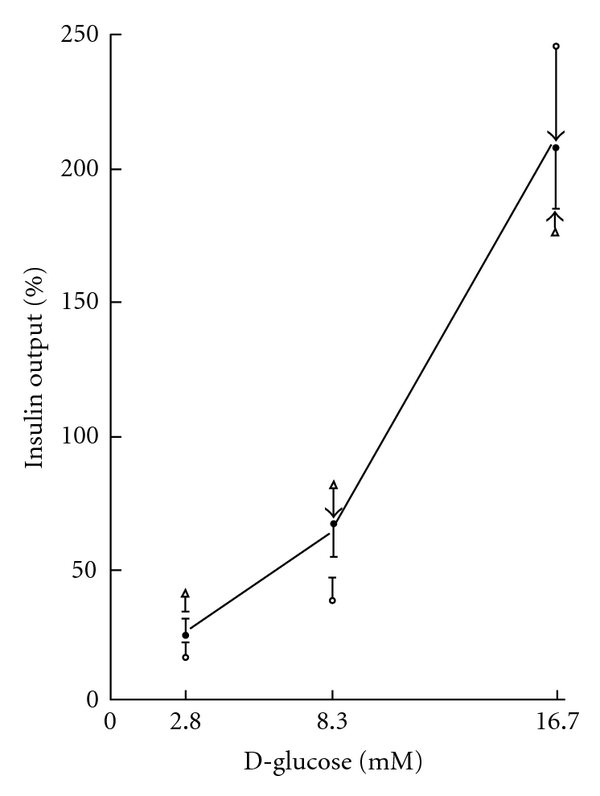
Insulin output by islets from NF control rats (open circles), IF control rats (closed circles), and CR control rats (open triangles) incubated at increasing concentrations of D-glucose. All results are expressed relative to the overall mean values recorded at the three concentrations of the hexose in each type of rats. Mean values (±SEM) refer to 10 (NF and CR rats) or 20 (IF rats) separate measurements, the SEM bar ending by an arrow whenever it exceeded the space to the next mean value. The solid line refers to the overall mean value recorded in the three groups of rats at each hexose concentration.

The release of insulin by islets prepared from control rats and incubated for 90 min at 8.3 mM D-glucose averaged 87.3 ± 12.7 *μ*U/islet (*n* = 40). As illustrated in Figure 5, the concentration-response relationship for insulin output at increasing concentration of the hexose was comparable in NF, IF, and CR control rats. At the most, there was a trend towards higher mean values in islets prepared from IF and CR control rats as distinct from NF control rats and incubated at 2.8 mM and 8.3 mM D-glucose. However, a significant difference between the mean values recorded at each D-glucose concentration in each type of control rats (NF, IF, and CR) was only observed once (*P* < 0.02) among nine comparisons.

The final insulin content of the islets prepared from control rats failed to differ significantly after incubation at 2.8, 8.3, or 16.7 mM D-glucose. Pooling all available data, it averaged, relative to the overall mean value recorded in each experiment after incubation at the three hexose concentrations, 96.0 ± 5.5% (*n* = 39), 98.8 ± 3.8% (*n* = 40), and 105.0 ± 3.3% (*n* = 40) in islets first exposed to 2.8, 8.3, and 16.7 mM, respectively. None of these mean values differed significantly from one another. Likewise, no significant difference was observed between the mean values for the insulin content of the islets prepared from NF control rats (347.2 ± 18.0 *μ*U/islet; *n* = 30) and either IF control rats (303.0 ± 14.0 *μ*U/islet; *n* = 30) or CR control rats (385.1 ± 16.4 *μ*U/islet; *n* = 29). In a further experiment conducted in IF control rats, the insulin content again failed to differ significantly from that recorded in the fed control rats after incubation at either 2.8 or 16.7 mM D-glucose. Only the mean value found in this further experiment in the islets from IF control rats after incubation at 8.3 mM happened to be lower (*P* < 0.01) than that otherwise recorded under the same experimental condition in the fed control rats.

 The release of insulin (*μ*U/islet per 90 min) by islets prepared from STZ rats averaged, at 2.8, 8.3, and 16.7 mM D-glucose, respectively, 2.08 ± 0.91 (*n* = 28), 4.01 ± 1.01 (*n* = 36), and 7.18 ± 1.70 (*n* = 36). It was thus significantly higher (*P* < 0.02) at 16.7 mM D-glucose than at 2.8 mM D-glucose. As judged from the mean values for insulin output and content measured in each experiment, the release of insulin represented 32.7 ± 11.7% (*n* = 4), 43.0 ± 19.4% (*n* = 5), and 62.6 ± 32.1% (*n* = 5) of the final insulin content of the islets after incubation at 2.8, 8.3, and 16.7 mM D-glucose, respectively. The insulin content of the islets prepared from STZ rats, expressed relative to the mean value found in each experiment in islets first incubated at 8.3 and 16.7 mM D-glucose (9.7 ± 1.6 *μ*U/islet; *n* = 70), averaged 165.5 ± 27.8% (*n* = 28) after exposure to 2.8 mM D-glucose, 105.1 ± 20.1% (*n* = 35) after exposure to 8.3 mM D-glucose, and 94.8 ± 17.4% (*n* = 35) after exposure to 16.7 mM D-glucose. Such a progressive decrease in insulin content as a function of the concentration of the hexose during incubation was validated by the significant difference (*P* < 0.03) found between the highest and lowest of these three percentages. In these respects, no significant difference was observed between NF, IF and CR diabetic animals.

 Both the insulin output and islet insulin content were dramatically lower in STZ rats than in control animals. For instance, over 90 min incubation at 8.3 mM D-glucose, the mean insulin output by islets from STZ rats did not exceed 4.01 ± 1.01 *μ*U/islet (*n* = 36), as distinct from a mean value of 87.3 ± 12.7 *μ*U/islet (*n* = 40) in control animals. Likewise, after 90 min incubation at 2.8 mM D-glucose, the insulin content of the islets did not exceed 5.9 ± 1.3 *μ*U/islet (*n* = 28) in STZ rats, as distinct (*P* < 0.001) from 331.5 ± 20.8 *μ*U/islet (*n* = 29) in control animals.

### 3.6. Islet Immunochemistry

**Figure 6:**
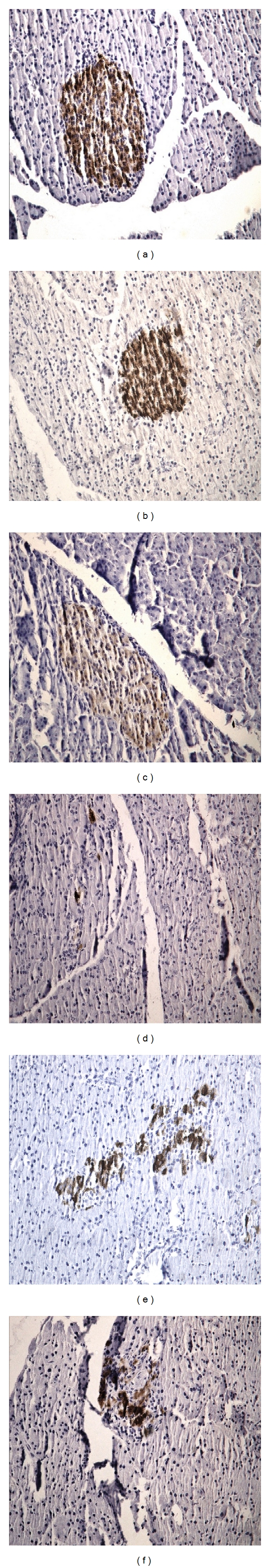
Immunodetection of insulin by the ABC-DAB technique in NF (a, d), IF (b, e), and CR (c, f) control (a, b, c) and STZ (d, e, f) rats.

**Figure 7:**
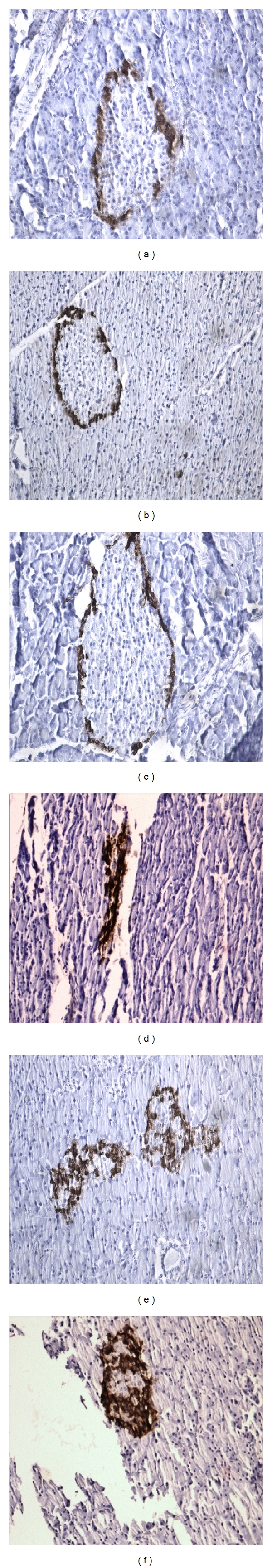
Immunodetection of glucagon by the ABC-DAB technique in NF (a, d), IF (b, e), and CR (c, f) control (a, b, c) and STZ (d, e, f) rats.

The detection of insulin-producing cells by the ABC-DAB technique yielded comparable images in NF, IF and CR control rats (Figures 6(a), 6(b), and 6(c)). In the STZ rats, however, the same technique revealed a severe decrease in insulin staining, such a decrease being apparently most pronounced in the NF animals (Figures 6(d), 6(e), and 6(f)). The immunodetection of glucagon-producing cells, by a comparable ABC-DAB technique is illustrated in Figure 7. In the NF, IF, and CR control rats, the glucagon-producing cells were typically located at the periphery of the islets. In the STZ rats, however, an apparently increased number of glucagon-producing cells seemed to invade the center of the islets.

The relative value occupied by *β*-cells in serial sections of the whole pancreas did not exceed 0.20 ± 0.05% (*n* = 9) in STZ rats, as compared (*P* < 0.001) to 1.06 ± 0.07% (*n* = 9) in control animals. The values recorded in the IF and CR control rats did not differ significantly (*P* > 0.23) from those found in the NF control rats (Table 5). In the STZ rats, however, both the relative and absolute values for *β*-cell mass were higher (*P* < 0.07 or less) in IF than NF animals, such not being the case when comparing CR and NF STZ rats. When multiplied by the weight of the pancreas measured after 5 min evaporation of formol, the total *β*-cell mass appeared somewhat lower in IF control rats (5.7 ± 1.3 mg; *n* = 3) than in either NF control rats (11.1 ± 1.5 mg; *n* = 3; *P* < 0.05) or CR control rats (9.6 ± 1.0 mg; *n* = 3; *P* < 0.08), with an overall mean value (8.8 ± 1.0 mg; *n* = 9) one order of magnitude higher (*P* < 0.001) than that recorded in the STZ rats (1.5 ± 0.4 mg; *n* = 9). In these experiments, the mean pancreatic weight failed to differ significantly (*P* > 0.66) in control animals (0.84 ± 0.09 g; *n* = 9) and STZ rats (0.79 ± 0.04 g; *n* = 9). In both cases, however, and relative to the mean values found in NF animals (100.0 ± 2.8%; *n* = 6), those recorded in the IF rats (68.9 ± 7.5%; *n* = 6) were significantly lower (*P* < 0.02) than those measured in the CR rats (99.9 ± 8.0%; *n* = 6). 

 The latter data were in fair agreement with the direct measurement of pancreas wet weight at sacrifice with a paired ratio between the values obtained by direct measurement at sacrifice and those reached after fixation averaging 97.7 ± 4.2% (*n* = 17). Even when expressed relative to body weight, the pancreatic wet weight at sacrifice represented in the IF rats no more than 80.8 ± 5.5% (*n* = 6; *P* < 0.06) of that recorded in the CR rats (100.0 ± 6.6%; *n* = 7) of the same group (control or STZ rats).

 As indicated in Table 6, the individual *β*-cell area averaged in the STZ rats 135.8 ± 5.2% (*n* = 60; *P* < 0.001) of the mean corresponding value found in control animals (100.0 ± 2.3%; *n* = 60) exposed to the same feeding schedule (NF, IF, or CR). Whether in control rats or STZ rats, the mean *β*-cell area was significantly higher (*P* < 0.04 or less) in IF animals than in NF and/or CR animals. Thus, relative to the corresponding mean value found in the NF animals of the same group (control or STZ), that is, 100.0 ± 5.0% (*n* = 40), the values recorded in CR and IF animals averaged, respectively, 107.0 ± 3.1% (*n* = 40; *P* > 0.24) and 123.9 ± 4.3% (*n* = 40; *P* < 0.001), the latter mean value being also significantly higher (*P* < 0.003) than the former one.

 As also documented in Table 6, the individual islet area represented in STZ rats 14.8 ± 1.9% (*n* = 60; *P* < 0.001) of that found in the control animals (100.0 ± 4.5%; *n* = 60) exposed to the same feeding schedule (NF, IF or CR). In the control animals, such a mean islet area (expressed as mm² × 10³) was not significantly different in NF rats (22.6 ± 1.6; *n* = 20), IF rats (19.3 ± 1.9; *n* = 20), and CR rats (23.7 ± 1.5; *n* = 20). In the STZ animals, however, it increased from 1.34 ± 0.19 (*n* = 20) in the NF rats to 4.50 ± 0.69 (*n* = 20; *P* < 0.001) in the IF rats and to 5.07 ± 0.82 (*n* = 20; *P* < 0.001) in the CR rats, the latter two mean values failing to differ significantly (*P* > 0.59) from one another.

**Figure 8:**
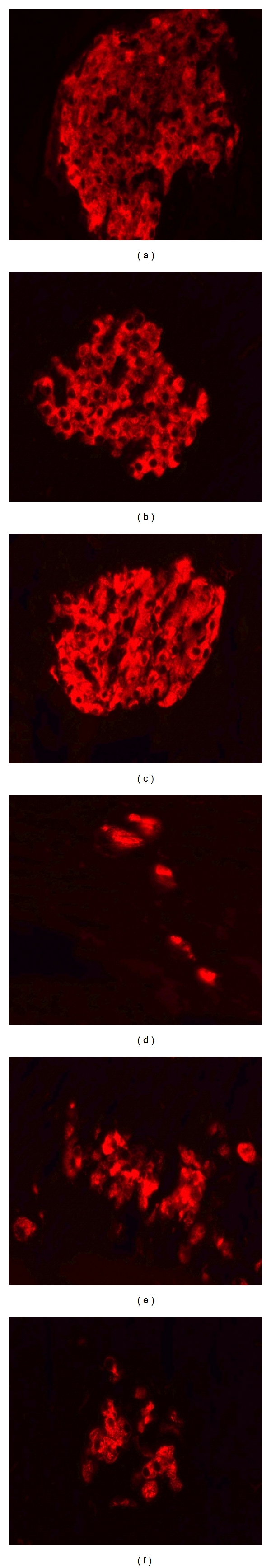
Immunodetection of insulin using rhodamine-conjugated secondary antibody in NF (a, d), IF (b, e), and CR (c, f) control (a, b, c) and STZ (d, e, f) rats.

**Figure 9:**
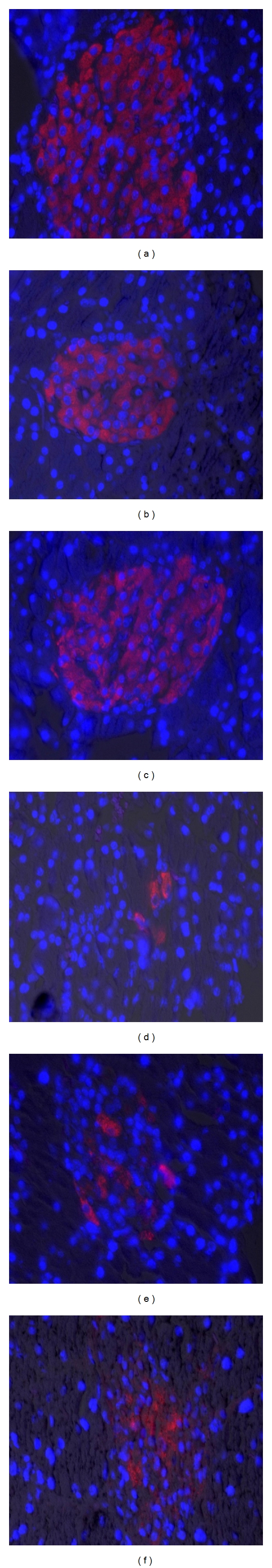
Detection of apoptotic *β*-cells by the TUNEL technique in insulin-stained pancreatic islet cells from NF (a, d), IF (b, e), and CR (c, f) control (a, b, c) and STZ (d, e, f) rats.

Figure 8 illustrates the immunodetection of *β*-cells using rhodamine-labelled secondary antibody, and Figure 9 the immunodetection of apoptotic *β*-cells by the TUNEL procedure. In the control animals, the percentage of apoptotic islet *β*-cells was comparable in NF, IF, and CR rats, with an overall mean value not exceeding 4.31 ± 0.10% (*n* = 15), as distinct (*P* < 0.001) from 14.31 ± 1.49% (*n* = 15) in STZ rats (Table 7). In the latter rats, the values recorded in IF and CR animals (11.45 ± 0.76%; *n* = 10) appeared lower (*P* < 0.004) than that found in the NF animals (20.03 ± 2.90%; *n* = 5), but the total number of *β*-cells examined in the NF STZ rats (101 ± 1 cells; *n* = 5) was much lower (*P* < 0.001) than that examined in the IF and CR rats (663 ± 62 cells; *n* = 10). Incidentally, even the latter value remained much lower (*P* < 0.001) than the total number of *β*-cells examined in the control animals (2, 236 ± 161 cells; *n* = 15). 

## 4. Discussion

In the light of a prior study conducted in sand arts [11–13], the major aim of the present experiments was to investigate the potential benefit of intermittent fasting in STZ-induced diabetic rats. Parallel experiments were here conducted in control animals.

As expected, the glycemia (or plasma D-glucose concentration) and the total AUC during an IPGTT were much higher in STZ rats than in control animals. Even the incremental AUC during the IPGTT was higher (*P* < 0.006) in STZ rats (608 ± 105 mM·min; *n* = 52) than in control animals (287 ± 29 mM·min; *n* = 48) despite a comparable initial increment in glycemia 30 min after the injection of D-glucose. Also, as expected, the plasma insulin concentration, the insulinogenic index, the secretion of insulin by isolated islets, their insulin content, the relative volume occupied by the *β*-cells in serial sections of the whole pancreas and the individual islet area were much lower in STZ rats than in control animals. Incidentally, a positive secretory response to D-glucose was still observed in isolated pancreatic islets from STZ rats, this coinciding with a progressive decrease in their final insulin content after incubation at increasing concentrations of D-glucose. Such a decrease was not observed, however, in pancreatic islets from control animals. Last, the HOMA for insulin resistance and percentage of apoptotic *β*-cells were also significantly higher in STZ rats than in control animals.

 In terms of morphological findings, there was, as a rule, little to distinguish between NF, IF, and CR control animals. At the most, there was a trend (*P* < 0.02) towards a higher individual *β*-cell area in IF than in NF control rats. Moreover, the pancreatic wet weight and, hence, total *β*-cell mass appeared lower (*P* < 0.04 or less) in IF control rats than in NF control rats. A comparable situation (*P* < 0.02) prevailed when comparing the pancreatic wet weight in IF STZ rats and NF STZ rats.

 In the STZ rats, the major other changes attributable to differences in feeding schedule concerned, in terms of morphological findings, the relative and absolute values for *β*-cell mass, the individual *β*-cell and islet area and the percentage of apoptotic *β*-cells. In the IF STZ rats, the relative and absolute values for *β*-cell mass, as well as the individual *β*-cell area and islet area, were all higher than in NF STZ rats, whilst the percentage of apoptotic cells appeared lower in IF than NF STZ rats. In the CR STZ rats, comparable findings were restricted to the individual islet area and percentage of apoptotic cells.

 The much higher percentage of apoptotic *β*-cells in STZ rats, as compared to control animals, is likely attributable to two major factors. First, the *β*-cell cytotoxic effect of STZ should not be ignored. According to Morimoto et al. [22], apoptosis of *β*-cells is already detected 6 hours after the injection of STZ, even before the onset of hyperglycemia. In this respect, it should be kept in mind that 3 to 14 days after STZ administration, infiltration of the islets by mononuclear cells takes place, eventually resulting in the removal of apoptotic cells by unspecific macrophages [23, 24]. Hence, the present data may well underestimate the apoptosis index otherwise prevailing during the first days after STZ administration. A second factor consists of the hyperglycemia resulting from STZ administration, since *in vitro* exposure of pancreatic islets to high concentrations of glucose also induces *β*-cell apoptosis [24].

 The latter process may account, in part at least, for the apparent differences between IF and CR STZ rats, in terms of both relative *β*-cell mass and individual *β*-cell area. Thus, according to the data listed in Tables 3 and 4, the glycemia (or plasma D-glucose concentration) after overnight starvation, expressed relative to the mean corresponding values found in NF STZ rats averaged in the IF STZ rats 64.9 ± 5.7% (*n* = 21), as distinct (*P* < 0.01) from 86.6 ± 5.6% (*n* = 23) in the CR STZ rats. The mean values for both the total and incremental AUC during the IPGTT were also higher in CR STZ rats than in IF STZ rats (Table 3). Such differences in glucose homeostasis coincided with higher mean values for both the relative volume of *β*-cells and their individual area in IF STZ rats as distinct from CR STZ rats (Tables 5 and 6). Thus, the mean relative value of *β*-cells was almost twice higher in IF than CR STZ rats, whilst the mean individual *β*-cell area represented in the IF STZ rats 125.4 ± 6.0% (*n* = 20; *P* < 0.002) of that found in CR STZ rats (100.0 ± 4.0%; *n* = 20). Despite the vastly different magnitude of the IF/CR ratio for these two variables, the difference between CR and IF STZ rats remained highly significant (*P* < 0.005) when pooling together the results recorded for each of these variables.

 The individual *β*-cell area was also always significantly higher in STZ rats than in control animals exposed to the same dietary schedule (Table 6). These converging findings concerning differences in individual *β*-cell area as a function of glucose tolerance are reminiscent of the hypertrophy of *β*-cells found either *in vitro* after exposure to a high concentration of D-glucose [25] or *in vivo* in rats which became hyperglycemic after partial pancreatectomy [26] and currently ascribed to a compensatory mechanism in residual *β*-cells no more susceptible to undergo mitosis [27]. 

 The latter consideration is not meant to deny that in addition to *β*-cell hypertrophy, an increase in *β*-cell number, possibly attributable to transdifferentiation of glucagon-producing to insulin-producing cells [28, 29], may participate in the difference in relative or total *β*-cell mass between NF and IF STZ rats, as also suggested by the total cell numbers listed in Table 7.

 A beneficial effect of intermittent fasting from 5 p.m. to 8 a.m. in STZ rats was documented by a decrease in glycemia at time zero of the IPGTT, a decrease in the total glycemic AUC during the IPGTT, a lower plasma D-glucose concentration at sacrifice after overnight starvation, and a trend towards a higher plasma insulin concentration and insulinogenic index and a lower HOMA index at sacrifice. For the latter three variables, the geometric means of the relevant variable (plasma insulin concentration, insulinogenic index, and inverse of HOMA index) yielded a significant difference (*P* < 0.025) between NF (100.0 ± 6.9%; *n* = 15) and IF (164.4 ± 29.5%; *n* = 18) STZ rats. Furthermore, no significant adverse effect of intermittent fasting (*P* > 0.49) was observed in terms of the changes in body weight of the STZ rats over the 30 days final experimental period, when comparing NF animals (−12.4 ± 2.8 g; *n* = 5) to IF rats (−18.8 ± 7.9 g; *n* = 6).

 A different situation prevailed in the calorie-restricted STZ rats. No statistically significant beneficial effects of caloric restriction in the STZ rats was observed when comparing NF to CR diabetic animals. Moreover, the decrease in body weight observed in the STZ rats during the final 30 days experimental period was 2.5 to 3.8 times higher (*P* < 0.001) in CR rats than in IF and NF animals, respectively.

 Even in control rats, the gain in body weight was much lower in CR animals than in IF ones (Figure 1). This coincided with lower mean values for the plasma insulin concentration, insulinogenic index, and HOMA index in IF control rats than in CR control rats examined at sacrifice after overnight starvation (Table 4). Thus, for these three variables, the values recorded in IF control rats averaged 71.6 ± 8.6% (*n* = 15; *P* < 0.06) of the mean corresponding values found in CR control rats (100.0 ± 11.5%; *n* = 15). Since such distinctions between IF and CR control rats could not be ascribed to any difference in either food intake or the responsiveness to D-glucose of isolated pancreatic islets incubated *in vitro*, they suggest a more stressful situation in CR control rats than in IF control animals. To a large extent, a comparable situation may prevail in CR as distinct from IF diabetic animals.

 In conclusion, therefore, the present study allows to extend to streptozotocin-induced diabetic rats, the proposal that intermittent fasting exerts a beneficial effect on glucose tolerance [11–13]. In our opinion, such a dietary approach merits to be also considered as a possible approach to prevent or minimize, if not correct, disturbances of glucose homeostasis in human subjects.

## Figures and Tables

**Table 1:** Food intake (g/day per rat).

Rats		Control period (last 6 days)^a^	Experimental period (30 days)^b^
Control	NF		20.1 ± 0.3 (5)
	IF		15.3 ± 0.5 (5)
	CR	18.7 ± 0.1 (6)	15.0 ± 0.1 (6)

STZ	NF	44.5 ± 1.8 (5)	46.0 ± 0.7 (5)
	IF	42.9 ± 0.9 (6)	25.8 ± 0.6 (6)
	CR	44.2 ± 1.6 (6)	26.5 ± 1.0 (6)

^a^Each individual value represents the mean of 6 successive determinations.

^b^Each individual value represents the mean of 26 determinations.

**Table 2:** IPGTT glycemic data in control rats.

Rats		Day	Time zero (mM)	Total AUC (mM·min)	Incremental AUC (mM·min)	(*n*)
Control	NF	10	5.12 ± 0.59	1,027 ± 73	412 ± 120	(5)
		20	5.21 ± 0.27	1,177 ± 121	552 ± 104	(5)
		29	6.44 ± 0.38	998 ± 104	225 ± 77	(5)

Control	IF	10	5.79 ± 0.36	927 ± 28	233 ± 49	(6)
		20	6.16 ± 0.42	1,014 ± 49	275 ± 28	(5)
		29	7.50 ± 0.49	1,061 ± 97	161 ± 113	(5)

Control	CR	10	6.54 ± 0.32	1,081 ± 34	295 ± 52	(5)
		20	6.21 ± 0.30	1,044 ± 30	299 ± 42	(6)
		29	6.64 ± 0.74	959 ± 56	160 ± 75	(6)

**Table 3:** IPGTT glycemic data in STZ rats.

Rats	Time zero (mM)	Total AUC (mM·min)	Incremental AUC (mM·min)	(*n*)
NF	27.84 ± 1.17	3,681 ± 155	279 ± 153	(20)
IF	17.97 ± 1.85	2,825 ± 174	668 ± 149	(15)
CR	25.38 ± 1.70	3,988 ± 194	943 ± 205	(17)

**Table 4:** Plasma D-glucose and insulin concentrations at sacrifice.

Rats		Plasma D-glucose (mM)	Plasma insulin (*μ*U/mL)	Insulinogenic index (mU/mmol)	HOMA (mM·*μ*u/mL)
Control	NF	8.61 ± 0.88 (5)	38 ± 6 (5)	8.63 ± 2.08 (5)	336 ± 73 (5)
	IF	8.12 ± 1.08 (5)	32 ± 2 (5)	9.44 ± 3.42 (5)	264 ± 45 (5)
	CR	7.80 ± 0.72 (6)	45 ± 2 (5)	14.61 ± 4.51 (5)	336 ± 68 (5)

STZ	NF	35.80 ± 2.47 (5)	16 ± 2 (5)	0.48 ± 0.08 (5)	548 ± 57 (5)
	IF	22.91 ± 4.30 (6)	22 ± 5 (6)	1.17 ± 0.51 (6)	419 ± 45 (6)
	CR	26.35 ± 4.20 (6)	25 ± 5 (6)	1.16 ± 0.52 (6)	592 ± 129 (6)

**Table 5:** Relative and absolute values for total *β*-cell mass.

Rats		Relative volume	Pancreas weight	*β*-cell mass
		(‰)	(g)	(mg)
Control	NF	10.76 ± 0.73 (3)	1.03 ± 0.06 (3)	11.12 ± 1.46 (3)
Control	IF	10.39 ± 2.04 (3)	0.55 ± 0.05 (3)	5.67 ± 1.28 (3)
Control	CR	10.56 ± 0.70 (3)	0.92 ± 0.13 (3)	9.58 ± 0.95 (3)
STZ	NF	1.11 ± 0.08 (3)	0.81 ± 0.01 (3)	0.90 ± 0.06 (3)
STZ	IF	3.71 ± 0.88 (3)	0.68 ± 0.03 (3)	2.55 ± 0.64 (3)
STZ	CR	1.31 ± 0.55 (3)	0.88 ± 0.06 (3)	1.20 ± 0.57 (3)

**Table 6:** Individual *β*-cell and islet area.

Rats		Sample	Individual *β*-cell area (*μ*m²)	Individual islet areas (mm² ×10³)
Control	NF	1	116.2 ± 3.0 (10)	23.72 ± 2.67 (10)
		2	102.4 ± 4.5 (10)	21.49 ± 1.85 (10)
Control	IF	1	123.6 ± 8.5 (10)	24.15 ± 3.13 (10)
		2	130.9 ± 8.7 (10)	14.50 ± 1.50 (10)
Control	CR	1	119.5 ± 8.3 (10)	23.30 ± 2.69 (10)
		2	117.3 ± 6.7 (10)	24.12 ± 1.61 (10)
STZ	NF	1	157.1 ± 25.2 (10)	1.11 ± 0.23 (10)
		2	131.1 ± 11.9 (10)	1.57 ± 0.30 (10)
STZ	IF	1	192.5 ± 14.0 (10)	3.34 ± 0.55 (10)
		2	186.4 ± 12.1 (10)	5.63 ± 1.18 (10)
STZ	CR	1	145.4 ± 8.5 (10)	5.94 ± 1.13 (10)
		2	156.8 ± 8.4 (10)	4.20 ± 1.18 (10)

**Table 7:** Number and percentage of apoptotic *β*-cells.

Rats		TUNEL plus cell number	Total cell number	Apoptotic *β*-cells (%)
Control	NF	113 ± 14 (5)	2,452 ± 325 (5)	4.65 ± 0.21 (5)
Control	IF	70 ± 7 (5)	1,732 ± 152 (5)	4.02 ± 0.10 (5)
Control	CR	107 ± 12 (5)	2,525 ± 219 (5)	4.26 ± 0.07 (5)
STZ	NF	19 ± 2 (5)	101 ± 17 (5)	20.03 ± 2.90 (5)
STZ	IF	68 ± 5 (5)	643 ± 66 (5)	10.68 ± 0.38 (5)
STZ	CR	90 ± 23 (5)	683 ± 113 (5)	12.22 ± 1.46 (5)
